# Supplementary material for: A context-dependent switch from sensing to feeling in the primate amygdala
Source: Cell Rep. Author manuscript; Available in PMC 2023 Oct 23. (PMC10430631; doi:10.1016/j.celrep.2023.112056)
Supplement: 1 [file NIHMS1921213-supplement-1.pdf]

Cell Reports, Volume *42*

Supplemental information

A context-dependent switch from sensing to feeling in the primate amygdala

Anne B. Martin, Michael A. Cardenas, Rose K. Andersen, Archer I. Bowman, Elizabeth A. Hillier, Sliman Bensmaia, Andrew J. Fuglevand, and Katalin M. Gothard

A context-dependent switch from sensing to feeling in the primate amygdala

Anne B. Martin[1], Michael A. Cardenas*[1], Rose K. Andersen*[1], Archer I. Bowman[1], Elizabeth A. Hillier, Sliman Bensmaia[2], Andrew J. Fuglevand[1], Katalin M. Gothard[1]

[1] The University of Arizona, College of Medicine, Depts. of Physiology and Neuroscience
[2] The University of Chicago, Dept. of Organismal Biology and Anatomy

* Michael A Cardenas and Rose Andersen contributed equally to the manuscript

Lead contact: Katalin M. Gothard, M.D., Ph.D.
kgothard@email.arizona.edu

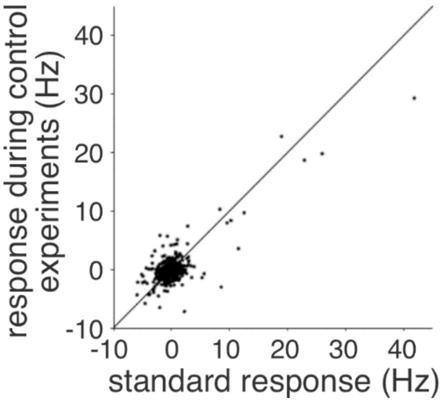

Figure S1. Control experiments, Related to Figure 3. Firing rate responses for 188 amygdala neurons recorded during control experiments plotted as a function of the responses during standard conditions. Control experiments involved changes in grooming pressure (n = 18), blindfolding (n = 40), changing reward contingencies (n = 40), changing predictability of stimuli (n = 36), and having the groomer sit in the booth during airflow blocks (n = 54). The responses during the standard experiment were highly correlated with the responses during the controls (Pearson correlation rho = 0.78, $p < 0.0001$). Therefore, manipulating various aspects of the experiment protocol did not influence the responses of the recorded neurons.

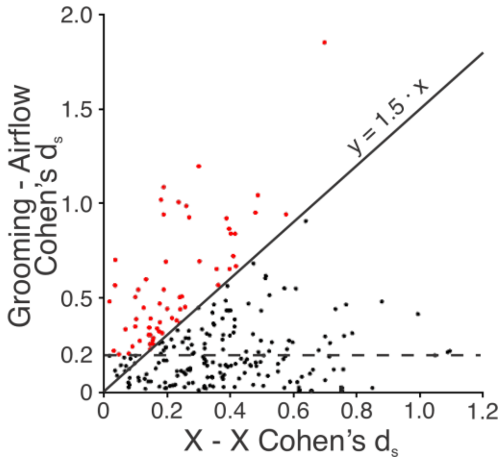

Figure S2. Identification of context-related changes in baseline firing rate, Related to Figure 4. Effect size for grooming-to-airflow comparison in baseline firing rates for 237 amygdala neurons plotted as a function of the largest effect size of either the grooming-to-grooming or airflow-to-airflow block comparisons (X-X). Dashed horizontal line represents the minimum effect size for grooming – airflow comparisons. Diagonal line represents 1.5 times the X-X effect size. For a neuron to be considered as exhibiting context-related activity in baseline firing, the grooming-to-airflow effect size needed to exceed the minimal effect-size threshold ($d_s = 0.2$) and be 1.5 times greater than the largest of the within condition (X-X) measures (red dots, n = 60). The mean (± SD) difference in baseline firing rates for these 60 cells was 1.2 ± 1.2 Hz.

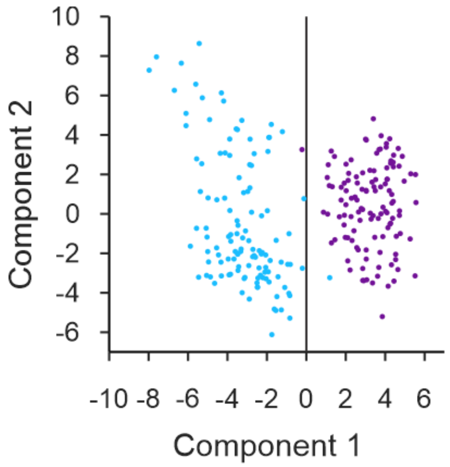

Figure S3. Context can be decoded from baseline firing rates despite minimal explained variance, Related to Figure 6. Principal component and k-means clustering of population baseline firing rates from grooming (purple) and airflow (blue) time bins. K-means clustering of the first component values into grooming or airflow time bins was accurate for 95.4% of bins, indicated by the clustering boundary at 0 (black line). The first component explained 8% of the overall baseline firing rate variance, corresponding to an average of 0.5 Hz difference in baseline firing rates between grooming and airflow blocks.

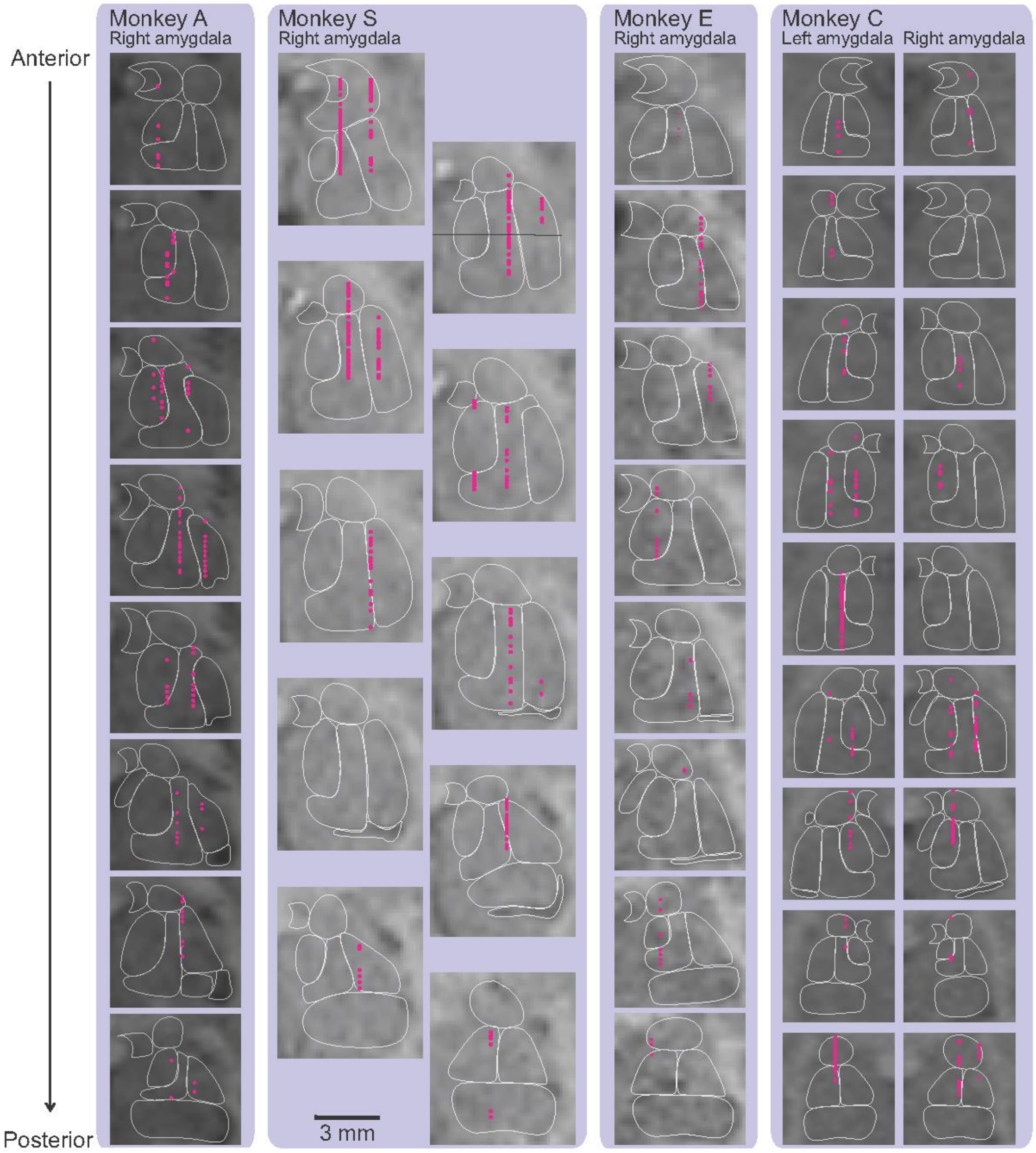

Figure S4. Anatomical reconstruction of amygdala recording sites for four monkeys, Related to Electrophysiological procedures in STAR Methods. From the structural MRI scans (isotropic voxel size of 0.5mm), an atlas was built for each monkey. The atlas contained all MRI slices that contained the amygdala. MRI slices are shown from most anterior (top) to most posterior (bottom). White lines indicate the estimated boundary of amygdala nuclei. Anatomical reconstructions of electrode targets were based on post-surgical MRIs that used columns of contrast positioned coaxially with the recording chambers, allowing us to calculate the x-y-z location of each recording site relative to the chamber coordinates (error magnitude maximum 1 mm). Red dots indicate the estimated locations of electrode contacts that recorded neurons included in this study.

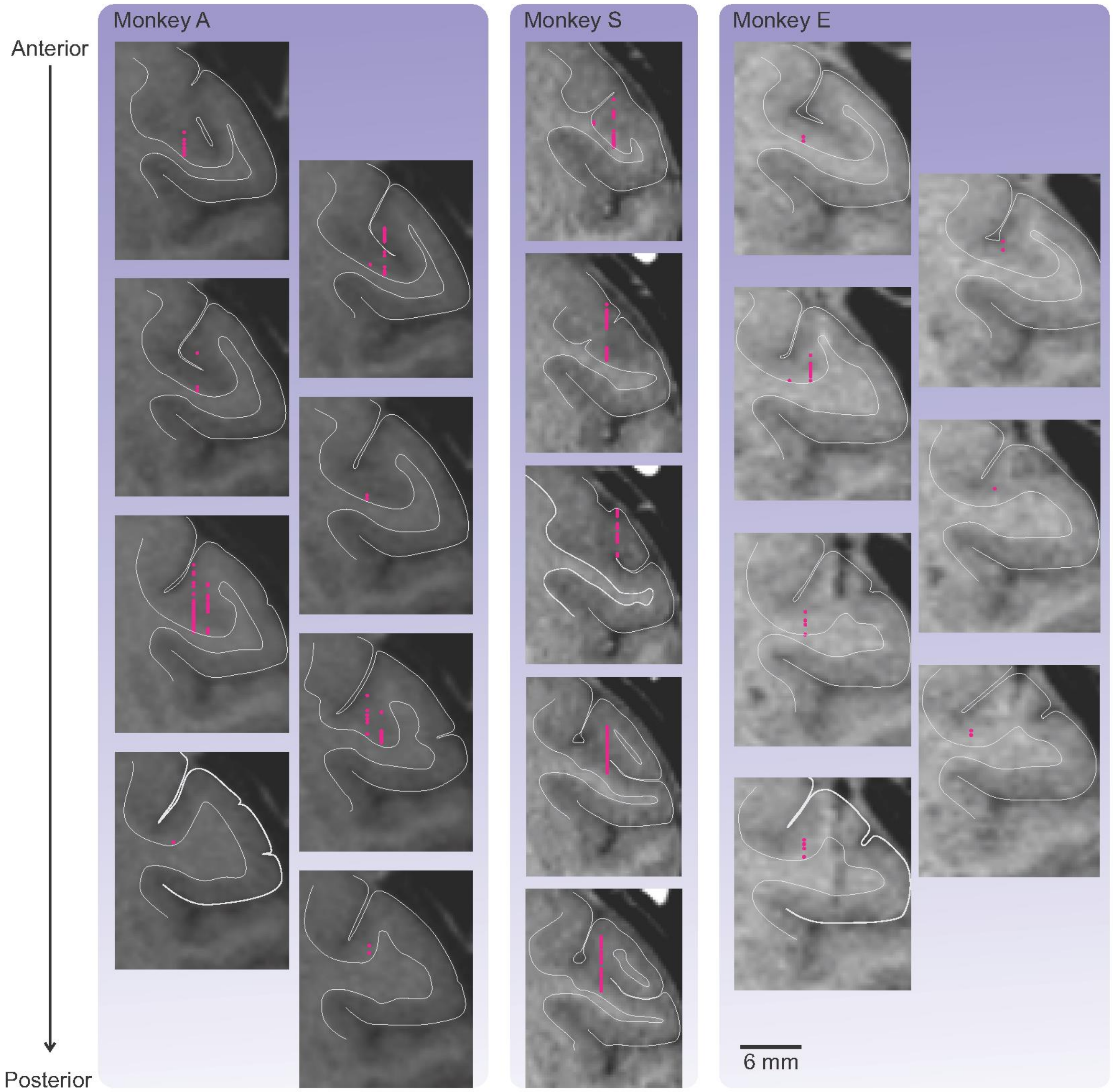

Figure S5. Anatomical reconstruction of SI recording sites for three monkeys, Related to Electrophysiological procedures in STAR Methods. MRI slices are shown from most anterior (top) to most posterior (bottom). Red dots indicate the estimated locations of electrode contacts that recorded neurons included in this study.
